# Drone-Fleet-Enabled Logistics: A Joint Design of Flight Trajectory and Package Delivery

**DOI:** 10.3390/s22083056

**Published:** 2022-04-15

**Authors:** Yunjian Jia, Yi Zhang, Kun Luo, Wanli Wen

**Affiliations:** 1 School of Microelectronics and Communication Engineering, Chongqing University, Chongqing 400044, China; yunjian@cqu.edu.cn (Y.J.); never_zy@cqu.edu.cn (Y.Z.); 20173825@cqu.edu.cn (K.L.); 2 National Mobile Communications Research Laboratory, Southeast University, Nanjing 210096, China

**Keywords:** logistics, drone fleet, package delivery, trajectory optimization, no-fly zone constraints

## Abstract

In this work, we focus on a drone-fleet-enabled package delivery scenario, in which multiple drones fly from a start point and cooperatively deliver packages to the ground users in the presence of a number of no-fly zones (NFZs). We first mathematically model the package delivery scenario in a rigorous manner. Then, a package value maximization problem is established to optimize the flight trajectory and package delivery under the constraints of drone load and collision as well as NFZs. The formulated problem is a highly challenging mixed-integer non-convex problem. To facilitate solving it, we transform the formulated problem into an equivalent problem with special structure by using some appropriate transformations, based on which a low-complexity algorithm with favorable performance is developed using the penalty convex–concave procedure method. Finally, numerical results demonstrate the superiority of the proposed solution.

## 1. Introduction

Over the past few years, drones have attracted extensive attention in the fields of wireless communications, the internet of things, logistics, and so on, due to their high mobility, autonomy, and flexibility [1]. In a wide variety of drone applications, the flight trajectory design of the drone is a pivotal but challenging task, since it is required not only to prevent collisions between drones, but also to avoid no-fly zones (NFZs), such as military bases, government agencies, and airports [2]. To tackle this challenging task, some representative research efforts have emerged in recent years, such as [3,4,5,6,7]. Specifically, the authors of [3,4] studied the problem of minimizing drone power consumption through the joint design of wireless resource management and path planning under NFZ constraints in drone-enabled communication systems. In [5], by carefully designing the drone trajectory in the presence of multiple NFZs, the authors maximized the uplink sum rate among all ground users. Note that only a single-drone scenario was examined in [3,4,5]. For a multi-drone scenario (i.e., drone fleet), the authors of [6,7] studied secure drone-enabled communication systems constrained by NFZs and collision avoidance, in which the system confidentiality was maximized by carefully planning the drone fleet trajectory.

We remark that the drones in the above literature [3,4,5,6,7] were only used as flying base stations to enhance the performance of the existing terrestrial wireless networks. However, as pointed out by [8], the use of drones for package delivery is increasingly becoming a new industry trend. In the drone delivery scenario, drones may not be able to deliver packages for all users at the same time due to load constraints, which therefore calls for a joint design of the flight trajectory and package delivery. Recently, only several works such as [9,10] have focused on the trajectory design in a drone delivery scenario, with the goal of minimizing the propulsion energy consumed by a single drone [9] or the flight path length of a drone fleet [10]. However, in [9,10], the authors implicitly assumed that there were no NFZs in the network, and did not take into account drone load limits, which obviously is not applicable for the real world. As a result, when considering the NFZ and load constraints, how to jointly design the flight trajectory and package delivery of drones has still not been tackled appropriately, which motivates this work.

Our main contributions can be summarized as follows. First, we conduct rigorous mathematical modeling of the drone-enabled package delivery scenario, in which a fleet of drones takes off from a start point and cooperatively delivers packages to users on the ground in the presence of multiple NFZs. Then, we establish a package value maximization problem by jointly optimizing the flight trajectory and package delivery under the constraints of drone load and collision as well as NFZs. The formulated problem is a highly challenging mixed-integer non-convex problem. To facilitate solving it, we transform the formulated problem into an equivalent problem with special structure by using some appropriate transformations. On this basis, we propose a low-complexity algorithm with favorable performance based on the penalty convex–concave procedure method. Finally, numerical results demonstrate the superiority of the proposed solution.

## 2. System Model

**Figure 1:**
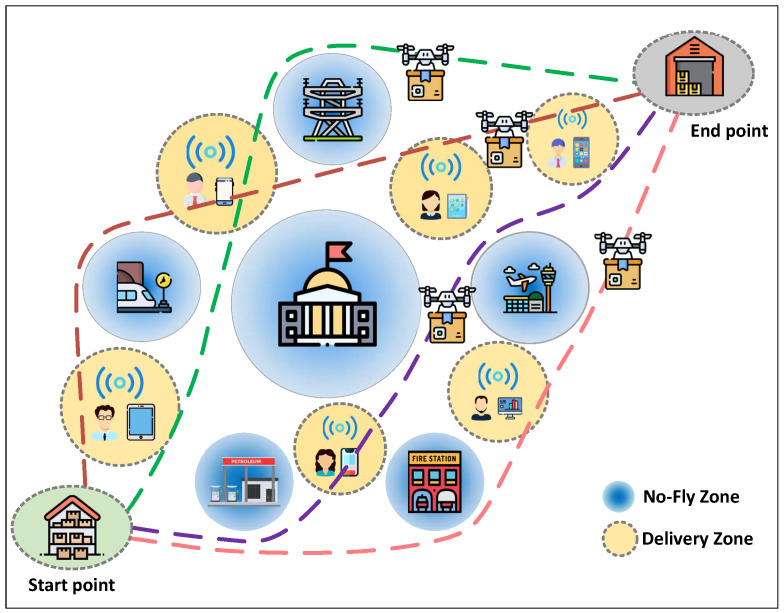
The schematic diagram of the system model. Source: elaborated by authors.

Let us consider a package delivery scenario using a drone fleet, as shown in Figure 1, in which there are *U* drones flying from the start point to the end point, aiming to deliver packages to *K* ground users within *T* time slots. For ease of illustration, let us define U≜{1,2,⋯,U}, K≜{1,2,⋯,K}, and T≜{1,2,⋯,T} to be the drone set, the user set, and the time slot set, respectively. Let $c_{u}\left[t\right]\in R^{2\times 1}$ represent the horizontal location of the *u*-th drone in slot t∈T. Then, to avoid collision between drones, we should have

(1) $$\begin{array}{cc} \; & ∥c_{u}\left[t\right]-c_{\tilde{u}}\left[t\right]∥\geq L^{\min},\;t\in T,\;u\in U,\;\tilde{u}\in U, \end{array}$$

(2) $$\begin{array}{cc} \; & c_{u}\left[1\right]=c^{\mathrm{str}},\;c_{u}\left[T\right]=c^{\mathrm{end}}, \end{array}$$

where $L^{\min}$ is the minimum distance between drones in each slot, and $c^{\mathrm{str}}\in R^{2\times 1}$ and $c^{\mathrm{end}}\in R^{2\times 1}$ are the horizontal locations of the start and end points, respectively. In addition, to bound the flight speed of drone *u*, we further have

(3) $$\begin{array}{c} ∥c_{u}\left[t\right]-c_{u}\left[t-1\right]∥\leq L_{u}^{\max},\;\;u\in U,\;t\in T\backslash \left\{1\right\}, \end{array}$$

where $L_{u}^{\max}$ is the *u*-th drone’s maximum traveling distance in a slot. We define $C≜{\left(C_{u}\right)}_{u\in U}$ with $C_{u}≜{\left(c_{u}\left[t\right]\right)}_{t\in T}$ as the trajectory design of the drone fleet.

We consider that there are *K* packages in the depot at the start point, and each package belongs to a different user. For ease of illustration, we refer to the package belonging to user *k* as package *k*. We assume that package *k* can be divided into $I_{k}\geq 1$ sub-packages, denoted by set $I_{k}≜\left\{1,2,\cdots ,I_{k}\right\}$. Each sub-package $i\in I_{k}$ has a weight of $w_{i,k}$ (in kilos) and thus the total weight of package *k* is given by $w_{k}≜\sum_{i\in I_{k}}w_{i,k}$. Note that each sub-package can be delivered by one drone at most and each package can be delivered cooperatively by multiple drones. Let $W_{u}$ (in kilos) denote the load limit of the *u*-th drone. Since different drones may have different load limits, we need to select a suitable number of drones for each package. Specifically, let $d_{i,k,u}\in \left\{0,1\right\}$ represent the delivery indicator variable of sub-package $i\in I_{k}$, where $d_{i,k,u}=1$ denotes that sub-package $i\in I_{k}$ is delivered by drone *u*, and $d_{i,k,u}=0$, otherwise. Then, we have the following constraints:

(4) $$\begin{array}{cc} \; & d_{i,k,u}\in \left\{0,1\right\},\;i\in I_{k},\;k\in K,\;u\in U, \end{array}$$

(5) $$\begin{array}{cc} \; & \sum_{u\in U}d_{i,k,u}\leq 1,\;i\in I_{k},\;k\in K, \end{array}$$

(6) $$\begin{array}{cc} \; & \sum_{u\in U}\sum_{i\in I_{k}}d_{i,k,u}\in \left\{0,I_{k}\right\},\;k\in K, \end{array}$$

where the constraint in (5) is due to the fact that each sub-package can only be delivered by one drone at most and the constraint in (6) comes from our stipulation that package *k* is delivered only when all sub-packages of package *k* are delivered. In addition, to prevent overload of drone *u*, the following constraint should be met:

(7) $$\begin{array}{cc} \; & \sum_{k\in K}\sum_{i\in I_{k}}d_{i,k,u}w_{i,k}\leq W_{u},\;u\in U. \end{array}$$

We define $D≜{\left(d_{i,k,u}\right)}_{i\in I_{k},k\in K,u\in U}$ as the package delivery design. Let $s_{k}\geq 0$ denote the value of the *k*-th package. Then, the total value of the packages delivered by the fleet can be expressed as $S\left(D\right)≜\sum_{k\in K}\frac{s_{k}}{I_{k}}\sum_{u\in U}\sum_{i\in I_{k}}d_{i,k,u}$. Note that in terms of the physical meaning, the value $s_{k}$ can represent the importance of package *k* or the priority of user *k*.

In order to deliver package *k*, the drone, such as the *u*-th drone, must fly to the delivery zone around user *k*, as depicted in Figure 1. We determine the delivery zone of user *k* according to whether drone *u* can detect the uplink signal of user *k*. As such, it is required to calculate the received signal-to-noise ratio at drone *u* during flight. As in [11], we assume that the channels from the users to the drones are only dominated by line-of-sight links. Then, we can compute the channel power gain from user *k* to drone *u* at slot *t* as $g_{k,u}\left[t\right]≜\frac{g_{0}}{h^{2}+{∥c_{u}\left[t\right]-a_{k}∥}^{2}}$, where $g_{0}$ denotes the channel power gain at a reference distance of one meter, $a_{k}\in R^{2\times 1}$ is the horizontal location of user *k*, and *h* is a constant, representing the altitude of each drone [1]. As a result, at drone *u*, the received signal-to-noise ratio from user *k* is given by $\gamma_{k,u}\left[t\right]=\frac{p_{k}g_{k,u}\left[t\right]}{N_{0}}$. Here, $p_{k}$ is the transmission power of user *k* and $N_{0}$ is the noise power. In view of the above notations, we now formally determine that the delivery zone of user *k* is the area where $\gamma_{k,u}\left[t\right]$ is greater than or equal to a pre-defined threshold $\theta_{u}$. Here, $\theta_{u}$ is related to the signal detection capability of drone *u*. Sub-package $i\in I_{k}$ can be delivered only when drone *u* arrives at the delivery zone of user *k*, so we have

(8) $$\begin{array}{cc} \; & d_{i,k,u}\leq \sum_{t\in T}𝟙\left[\gamma_{k,u}\left[t\right]\geq \theta_{u}\right],\;i\in I_{k},\;k\in K,\;u\in U, \end{array}$$

where 𝟙[·] represents an indicator function.

Finally, it should be noted that the drones are prohibited from crossing the NFZs when delivering packages, as illustrated in Figure 1. We assume that there exist *N* NFZs in the network, denoted by N≜{1,2,⋯,N}, and characterize the *n*-th NFZ as a cylindrical volume with a height greater than or equal to *h* [6]. We define $b_{n}\in R^{2\times 1}$ and $Z_{n}$ to be the horizontal coordinate center and radius of NFZ *n*, respectively. Then, to keep away from each NFZ, the following constraint should be satisfied:

(9) $$\begin{array}{c} ∥c_{u}\left[t\right]-b_{n}∥\geq Z_{n},\;n\in N,\;u\in U,\;t\in T. \end{array}$$

## 3. Problem Establishment

In this work, we would like to maximize the total value of the packages delivered by the fleet, i.e., S(D), through jointly optimizing the trajectory design C and the package delivery design D subject to the constraints in (1)–(9). Specifically, we have the following optimization problem.

(10) $$\begin{array}{cc} \max_{C,D} & \;S(D)\\ s.t.\;\; & \left(1)–(9\right). \end{array}$$

The problem in (10) is a challenging mixed-integer non-convex problem with the indicator function. To solve it in a computationally tractable manner, some necessary transformations are required, as detailed below.

To be specific, we first eliminate the indicator function in (8). Let $M_{k,u}\left[t\right]$ denote a large constant greater than any possible value of $∥c_{u}\left[t\right]-a_{k}{∥}^{2}$. Then, by utilizing the big-M approach and introducing a new binary variable $X≜{\left(x_{k,u}\left[t\right]\right)}_{k\in K,u\in U,t\in T}$, we can equivalently convert the constraint in (8) to

(11) $$\begin{array}{cc} \; & x_{k,u}\left[t\right]\in \left\{0,1\right\},\;k\in K,\;u\in U,\;t\in T, \end{array}$$

(12) $$\begin{array}{cc} \; & d_{i,k,u}\leq \sum_{t\in T}x_{k,u}\left[t\right],\;i\in I_{k},\;k\in K,\;u\in U, \end{array}$$

(13) $$\begin{array}{c} ∥c_{u}\left[t\right]-a_{k}{∥}^{2}\leq M_{k,u}\left[t\right]\left(1-x_{k,u}\left[t\right]\right)\\ \;\;+\frac{g_{0}p_{k}}{\theta N_{0}}-h^{2},\;k\in K,u\in U,\;t\in T. \end{array}$$

Second, by introducing a new binary variable $B≜{\left(b_{k}\right)}_{k\in K}$, we equivalently convert the constraint in (6) to

(14) $$\begin{array}{cc} \; & b_{k}\in \left\{0,1\right\},\;k\in K, \end{array}$$

(15) $$\begin{array}{cc} \; & b_{k}=\frac{1}{I_{k}}\sum_{u\in U}\sum_{i\in I_{k}}d_{i,k,u},\;k\in K. \end{array}$$

Third, we eliminate the binary variables D, X, and B. Using the similar method adopted in [12], we can equivalently transform the binary constraints in (4), (11) and (14) to the following continuous constraints:

(16) $$\begin{array}{cc} \; & d_{i,k,u}\in \left[0,1\right],\;i\in I_{k},\;k\in K,\;u\in U, \end{array}$$

(17) $$\begin{array}{cc} \; & x_{k,u}\left[t\right]\in \left[0,1\right],\;k\in K,\;u\in U,\;t\in T, \end{array}$$

(18) $$\begin{array}{cc} \; & b_{k}\in \left[0,1\right],\;k\in K, \end{array}$$

(19) $$\begin{array}{cc} \; & d_{i,k,u}\left(1-d_{i,k,u}\right)\leq 0,\;i\in I_{k},\;k\in K,\;u\in U, \end{array}$$

(20) $$\begin{array}{cc} \; & x_{k,u}\left[t\right]\left(1-x_{k,u}\left[t\right]\right)\leq 0,\;k\in K,\;u\in U,\;t\in T, \end{array}$$

(21) $$\begin{array}{cc} \; & b_{k}\left(1-b_{k}\right)\leq 0,\;k\in K. \end{array}$$

Last, based on the above transformations, the problem in (10) can be equivalently transformed to the following problem.

(22) $$\begin{array}{cc} \; & \max_{C,D,X,B}\;S\left(D\right)\\ s.t.\;\; & \left(1)–(3),(5),(7),(9),(12),(13),(15)–(21\right). \end{array}$$

Note that, the constraints in (1), (9) and (19)–(21) are non-convex. Fortunately, the left-hand sides of these constraints can be written as the difference of two convex functions. In the following section, we will see that this special structure greatly facilitates solving the problem in (22).

## 4. Low-Complexity Solution

**Figure 2:**
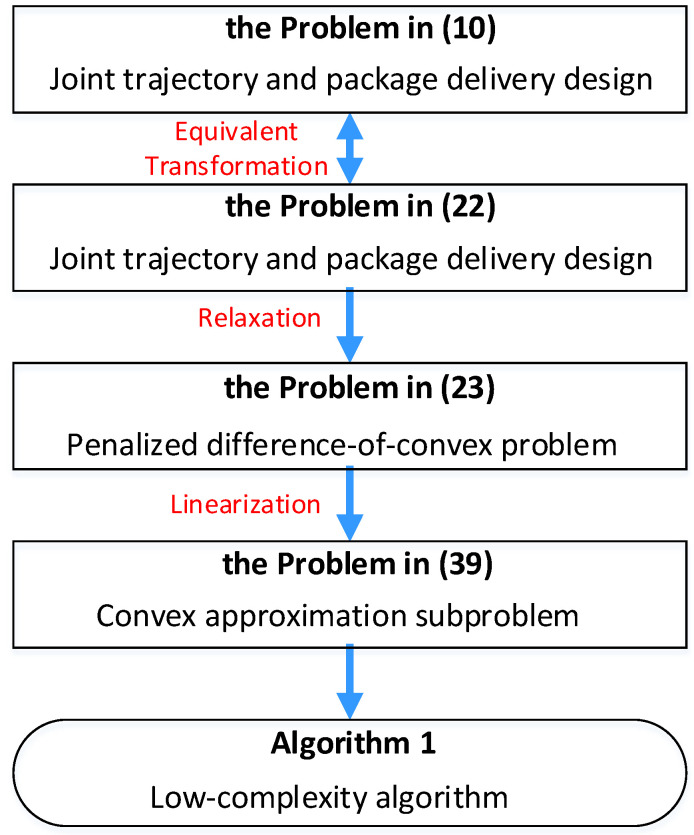
Flow chart for solving the problem in (10). Source: elaborated by authors.

Due to the special structure mentioned above, the problem in (22) can be regarded as a difference-of-convex problem. As such, we propose a low-complexity algorithm for solving the problem in (22) based on the penalty convex–concave procedure, which generally consists of two steps, namely relaxation and linearization. The overall procedure for solving the problem in (22) is shown in Figure 2.

### 4.1. Relaxation

In this step, by adding slack variables to (1), (9) and (19)–(21), and penalizing the sum of the corresponding violations, we obtain a penalized difference-of-convex problem as follows.

(23) $$\begin{array}{cc} \; & \max_{C,D,X,B,\varepsilon ,\sigma ,\alpha ,\beta ,\zeta }S\left(D\right)-\xi P\left(\varepsilon ,\sigma ,\alpha ,\beta ,\zeta \right)\\ s.t.\; & \left(2),(3),(5),(7),(12),(13),(15)–(18\right),\\ \; & -∥c_{u}\left[t\right]-c_{\tilde{u}}{\left[t\right]∥}^{2}\leq -{\left(L^{\min}\right)}^{2} \end{array}$$

(24) $$\begin{array}{cc} \; & \;\;\;+\varepsilon_{u,\tilde{u}}\left[t\right],\;u\in U,\;\tilde{u}\in U,\;t\in T,\\ \; & -∥c_{u}\left[t\right]-b_{n}{∥}^{2}\leq -Z_{n}^{2} \end{array}$$

(25) $$\begin{array}{cc} \; & \;\;\;+\sigma_{n,u}\left[t\right],\;n\in N,\;u\in U,\;t\in T, \end{array}$$

(26) $$\begin{array}{cc} \; & d_{i,k,u}\left(1-d_{i,k,u}\right)\leq \alpha_{i,k,u},\;i\in I_{k},\;k\in K,\;u\in U, \end{array}$$

(27) $$\begin{array}{cc} \; & x_{k,u}\left[t\right]\left(1-x_{k,u}\left[t\right]\right)\leq \beta_{k,u}\left[t\right],k\in K,u\in U,t\in T, \end{array}$$

(28) $$\begin{array}{cc} \; & b_{k}\left(1-b_{k}\right)\leq \zeta_{k},\;k\in K, \end{array}$$

(29) $$\begin{array}{cc} \; & \varepsilon_{u,\tilde{u}}\left[t\right]\geq 0,\;u\in U,\;\tilde{u}\in U,\;t\in T, \end{array}$$

(30) $$\begin{array}{cc} \; & \sigma_{n,u}\left[t\right]\geq 0,\;n\in N,\;u\in U,\;t\in T, \end{array}$$

(31) $$\begin{array}{cc} \; & \alpha_{i,k,u}\geq 0,\;i\in I_{k},\;k\in K,\;u\in U, \end{array}$$

(32) $$\begin{array}{cc} \; & \beta_{k,u}\left[t\right]\geq 0,\;k\in K,\;u\in U,\;t\in T, \end{array}$$

(33) $$\begin{array}{cc} \; & \zeta_{k}\geq 0,\;k\in K. \end{array}$$

Here,

$$\begin{array}{c} P\left(\varepsilon ,\sigma ,\alpha ,\beta ,\zeta \right)≜\sum_{t\in T}\sum_{u\in U}\sum_{\tilde{u}\in U}\varepsilon_{u,\tilde{u}}\left[t\right]+\sum_{t\in T}\sum_{n\in N}\sum_{u\in U}\sigma_{n,u}\left[t\right]+\\ \sum_{i\in I_{k}}\sum_{k\in K}\sum_{u\in U}\alpha_{i,k,u}+\sum_{t\in T}\sum_{k\in K}\sum_{u\in U}\beta_{k,u}\left[t\right]+\sum_{k\in K}\zeta_{k}, \end{array}$$

denotes the total violation, where $\varepsilon ≜{\left(\varepsilon_{u,\tilde{u}}\left[t\right]\right)}_{u\in U,\tilde{u}\in U,t\in T}$, $\sigma ≜{\left(\sigma_{n,u}\left[t\right]\right)}_{n\in N,u\in U,t\in T}$, $\alpha ≜{\left(\alpha_{i,k,u}\right)}_{i\in I_{k},k\in K,u\in U}$, $\beta ≜{\left(\beta_{k,u}\left[t\right]\right)}_{k\in K,u\in U,t\in T}$, and $\zeta ≜{\left(\zeta_{k}\right)}_{k\in K}$ are the slack variables for the constraints in (1), (9) and (19)–(21), respectively. The coefficient ξ>0 in the problem in (23) represents a penalty parameter and the increase in ξ will force the total violation P(ε,σ,α,β,ζ) to zero. In particular, if P(ε,σ,α,β,ζ)=0, the problems in (22) and (23) are equivalent.

### 4.2. Linearization

In this step, we linearize the concave terms in (24)–(28); that is, $-∥c_{u}\left[t\right]-c_{\tilde{u}}{\left[t\right]∥}^{2}$, $-∥c\left[t\right]-b_{n}{∥}^{2}$, $-{\left(d_{i,k,u}\right)}^{2}$, $-{\left(x_{k,u}\left[t\right]\right)}^{2}$, and $-{\left(b_{k}\right)}^{2}$, by applying the first-order Taylor expansion at the given point $\left(C^{\left(j\right)},D^{\left(j\right)},X^{\left(j\right)},B^{\left(j\right)}\right)$, where $C^{\left(j\right)}≜{\left(c_{u}^{\left(j\right)}\left[t\right]\right)}_{u\in U,t\in T}$, $D^{\left(j\right)}≜{\left(d_{i,k,u}^{\left(j\right)}\right)}_{i\in I_{k},k\in K,u\in U}$, $X^{\left(j\right)}≜{\left(x_{k,u}^{\left(j\right)}\left[t\right]\right)}_{k\in K,u\in U,t\in T}$, $B^{\left(j\right)}≜{\left(b_{k}^{\left(j\right)}\right)}_{k\in K}$, and j=1,2,⋯ denotes an iteration index. As such, at iteration *j*, the constraints in (24)–(28) can be approximated as the following linear constraints:

(34) $$\begin{array}{c} ∥c_{u}^{\left(j\right)}\left[t\right]-c_{\tilde{u}}^{\left(j\right)}{\left[t\right]∥}^{2}-2{\left(c_{u}^{\left(j\right)}\left[t\right]-c_{\tilde{u}}^{\left(j\right)}\left[t\right]\right)}^{T}\left(c_{u}\left[t\right]-c_{\tilde{u}}\left[t\right]\right)\\ \;\leq -{\left(L^{\min}\right)}^{2}+\varepsilon_{u,\tilde{u}}\left[t\right],\;u\in U,\;\tilde{u}\in U,\;t\in T, \end{array}$$

(35) $$\begin{array}{c} -∥c_{u}^{\left(j\right)}\left[t\right]-b_{n}{∥}^{2}-2{\left(c_{u}^{\left(j\right)}\left[t\right]-b_{n}\right)}^{T}\left(c_{u}\left[t\right]-c_{u}^{\left(j\right)}\left[t\right]\right)\\ \;\leq -Z_{n}^{2}+\sigma_{n,u}\left[t\right],\;n\in N,u\in U,\;t\in T, \end{array}$$

(36) $$\begin{array}{c} {\left(d_{i,k,u}^{\left(j\right)}\right)}^{2}+d_{i,k,u}\left(1-2d_{i,k,u}^{\left(j\right)}\right)\\ \;\leq \alpha_{i,k,u},\;i\in I_{k},\;k\in K,\;u\in U, \end{array}$$

(37) $$\begin{array}{c} {\left(x_{k,u}^{\left(j\right)}\left[t\right]\right)}^{2}+x_{k,u}\left[t\right]\left(1-2x_{k,u}^{\left(j\right)}\left[t\right]\right)\\ \;\leq \beta_{k,u}\left[t\right],\;k\in K,\;u\in U,\;t\in T, \end{array}$$

(38) $$\begin{array}{cc} \; & {\left(b_{k}^{\left(j\right)}\right)}^{2}+b_{k}\left(1-2b_{k}^{\left(j\right)}\right)\leq \zeta_{k},\;k\in K. \end{array}$$

Here, ${\left(\cdot \right)}^{T}$ represents the transpose operator. By replacing (24)–(28) with (34)–(38), we can obtain a convex approximation subproblem at iteration *j*, as detailed below.

(39) $$\begin{array}{cc} \; & \max_{C,D,X,B,\varepsilon ,\sigma ,\alpha ,\beta ,\zeta }S\left(D\right)-\xi^{\left(j\right)}P\left(\varepsilon ,\sigma ,\alpha ,\beta ,\zeta \right)\\ s.t.\;\; & \left(2),(3),(5),(7),(12),(13),(29)–(38\right). \end{array}$$

Here, $\xi^{\left(j\right)}$ is the penalty coefficient at iteration *j*. The problem in (39) is a convex optimization problem, which, therefore, can be solved optimally using existing software toolkits such as CVX.

### 4.3. Algorithm Summary

The detailed algorithm for solving the problem in (22) is summarized in Algorithm 1. Algorithm 1 begins with an arbitrary initial point $\left(C^{\left(0\right)},D^{\left(0\right)},X^{\left(0\right)},B^{\left(0\right)}\right)$ and a small penalty coefficient $\xi^{\left(0\right)}$, and then increases the penalty coefficient with a constant ν>1 at each iteration until a limit bound $\xi_{\max}$ is achieved to ensure P(ε,σ,α,β,ζ)→→0. It is worth mentioning that Algorithm 1 is able to converge to a stationary point of the problem in (22). The proof is similar to that in [13], which is omitted here for the sake of brevity. The complexity of Algorithm 1 is dominated by solving the problem in (39). If this problem is solved via CVX, the complexity is given by $O(A^{3.5}\log\left(1/\epsilon \right))$ [14], where A=UT(N+U+2K+2)+2K(U+1) and ϵ>0 represent the number of optimization variables and the given solution accuracy, respectively.
**Algorithm 1:** Low-complexity Algorithm for the problem in (22)1:Choose an arbitrary initial point $\left(C^{\left(0\right)},D^{\left(0\right)},X^{\left(0\right)},B^{\left(0\right)}\right)$, $\xi^{\left(0\right)}>0$, $\xi_{\max}$, and ν>1.2:Set j←0.3:**repeat**4:   Solve the problem in (39) to obtain an optimal solution $\left(C^{\left(j+1\right)},D^{\left(j+1\right)},X^{\left(j+1\right)},B^{\left(j+1\right)}\right)$.5:   Set the optimal value of the problem in (39) to ${\hat{V}}^{\left(j+1\right)}$.6:   Set $\xi^{\left(j+1\right)}\leftarrow \min\left\{\nu \xi^{\left(j\right)},\xi_{\max}\right\}$.7:   Set j←j+1.8:**until**$\frac{{\hat{V}}^{\left(j+1\right)}-{\hat{V}}^{\left(j\right)}}{{\hat{V}}^{\left(j\right)}}\leq 1\times 10^{-5}$ or j>50.

## 5. Numerical Results

To facilitate demonstrating the performance of our proposed scheme, we consider that the users and NFZs are randomly distributed in a 2D area of 1.2×1.2 ${\mathrm{km}}^{2}$. Unless otherwise mentioned, the simulation settings are given as follows: $c^{\mathrm{str}}={\left(-550,-550\right)}^{T}$, $c^{\mathrm{end}}={\left(550,550\right)}^{T}$, N=4, $I_{k}=1$, $p_{k}=0.01$ Watt for all k∈K, $W_{u}=55$ kilos, $\theta_{u}=-1.5$ dB, $L_{u}^{\max}=50$ meters for all u∈U, h=100, $L^{\min}=15$ meters, $g_{0}=10^{-6}$ dB, $N_{0}=10^{-12}$ Watt, $Z_{n}=150$ meters if *n* is odd and $Z_{n}=100$ meters, otherwise. In addition, we consider that the weight and the value of package k∈K follow a uniform distribution in the range [10,40] and each sub-package of package *k* has the same weight. For Algorithm 1, we randomly choose ν from (1.0,10] in each iteration and set $\xi^{\left(0\right)}=1\times 10^{-6}$ and $\xi_{\max}=1\times 10^{6}$.

**Figure 3:**
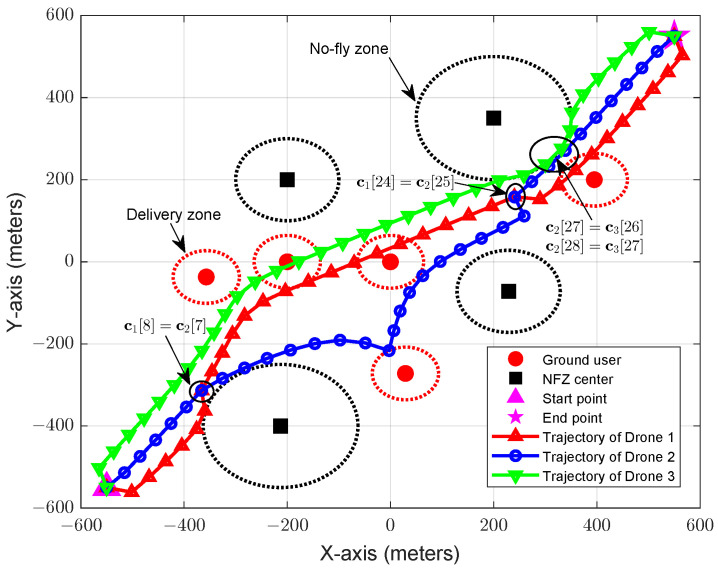
The trajectory of the drone fleet. Source: elaborated by authors.

Figure 3 shows the flight trajectory of the drone fleet, where we set T=35, K=5, U=3, and $L^{\min}=50$ m. From Figure 3, some observations can be made as follows. First, we can observe that each drone in the fleet does not fly into the NFZs, demonstrating that the proposed Algorithm 1 is able to effectively avoid the NFZs. Second, from their trajectory curves, we can easily verify that there are no collisions between drones. (It is noted that although the trajectory curves of drones are overlapped at some points, they are not overlapped at the same slot. For instance, for the first cross-point of drones 1 and 2, the horizontal coordinates of the two drones are $c_{1}\left[8\right]$ and $c_{2}\left[7\right]$, and for the second cross-point, the horizontal coordinates are $c_{1}\left[24\right]$ and $c_{2}\left[25\right]$, where $c_{1}\left[8\right]=c_{2}\left[7\right]$ and $c_{1}\left[24\right]=c_{2}\left[25\right]$. We can see that drones 1 and 2 actually do not meet at the same slot and thus, they will not collide.) Last, we can find that some drones (e.g., drones 1 and 3) will touch the users’ delivery zone, since they are required to perform package delivery tasks.

**Figure 4:**
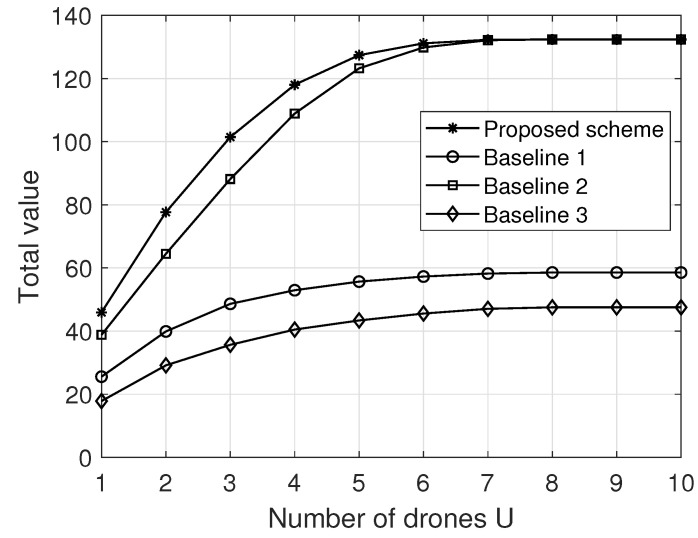
The total value versus the number of drones at $W_{u}=30$. Source: elaborated by authors.

**Figure 5:**
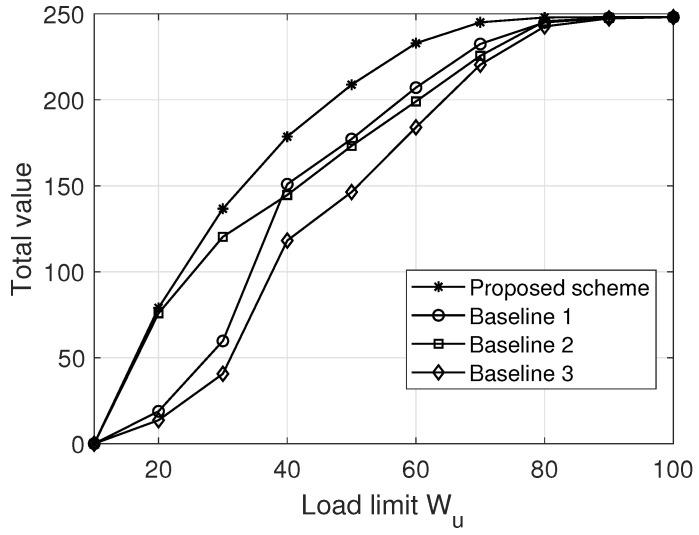
The total value versus the load limit at U=4. Source: elaborated by authors.

Figure 4 and Figure 5 compare our proposed scheme with some representative baselines at T=100 and K=10, where the results are averaged over 500 trials. To be specific, under the maximum load constraint in (7) the following baselines are considered. For Baseline 1, multiple packages with the highest value are delivered by the fleet. For Baseline 2, multiple lightest packages are delivered by the fleet. For Baseline 3, multiple packages closest to the end point are delivered by the fleet. Note that for each baseline, the flight trajectory of each drone can be achieved via Algorithm 1.

More specifically, Figure 4 and Figure 5 plot the total value S(D) versus the number of drones *U* and the load limit $W_{u}$, respectively. From Figure 4 and Figure 5, it first can be seen that the performance of all schemes improves as *U* ($W_{u}$) increases, due to the increase in fleet carrying capacity. However, with the increase in *U* ($W_{u}$), the marginal performance gain of adding more drones (storage capacity) into the fleet decreases, because the overall carrying capacity of the fleet is gradually large enough to carry all users’ packages. In addition, we also see that Baseline 2 significantly outperforms (slightly underperforms) Baseline 1 and Baseline 3 when $W_{u}$ is relatively small (large), e.g., $W_{u}\geq 40$ ($W_{u}<40$), indicating that the value- and distance-based schemes cannot work well in the area of each drone having a relatively small load limit. Finally, we can observe that the proposed scheme is superior to all baselines, because it can adapt well to the changes in the key system parameters through carefully optimizing the package delivery and fleet trajectory.

## 6. Discussion

Compared to the existing studies, the major innovativeness of our work lies in the following two aspects. The first point is that we focus on a drone-fleet-enabled package delivery scenario, in which multiple drones fly from a start point and cooperatively deliver packages to the ground users in the presence of a number of NFZs. This scenario is rarely considered in the current literature, but it is rigorously modeled mathematically in our paper. The second point is that by formulating a mixed-integer non-convex optimization problem and utilizing some appropriate transformation techniques (such as eliminating the indicator function and binary variables), we successfully develop a low-complexity algorithm based on the penalty convex–concave procedure method and achieve a joint design of drone fleet trajectory and package delivery.

The advantages of the joint design of drone fleet trajectory and package delivery proposed in this work lie in the following two aspects. On the one hand, under our design, the drone fleet can successfully deliver all packages of the ground users neither passing through the existing NFZs nor colliding with each other. On the other hand, the performance of our proposed joint design is better than the representative baselines due to its good adaptability to different system design parameters, such as the number of drones and the drone load limits, etc. These advantages suggest that the proposed joint design in this paper can address the challenges presented in Section 1 (i.e., the Introduction), and we believe that it can provide a theoretical reference for drone-enabled package delivery in real-world settings.

There are several interesting research directions worthy of extending the results of this paper in the future. (i) Energy-efficient drone-fleet-enabled package delivery: In this paper, we aim to maximize the total value of the packages delivered under the constraints of NFZs and the drone load, as well as the collision avoidance. We have not considered the energy consumption of drones. However, due to the limited capacity of the onboard battery of drones, the energy consumption of the drones needs to be considered when designing the flight trajectories of drones [15,16,17]. Thus, it is necessary to maximize the total value of delivered packages under the constraint of the energy consumption of the drone fleet. (ii) Optimal 3D trajectory of drone fleet: In this paper, we ignore the changes in the drone height in order to simplify the flight trajectory design of the drone fleet. However, each drone cannot only change its horizontal coordinate, but also its height; that is to say, the flying environment of the drone is 3D [18,19,20]. Therefore, in our future work, we are going to consider more realistic scenarios to optimize the 3D flight trajectory and package delivery design to maximize the total value of delivered packages.

## 7. Conclusions

In this paper, we studied a package delivery scenario supported by a drone fleet, in which a drone fleet flies from the start point in the presence of multiple NFZs and delivers packages to the ground users in a cooperative manner. We first rigorously modeled the package delivery scenario. Then, a package value maximization problem was established to optimize the flight trajectory and package delivery under the constraints of drone load and collision as well as NFZs. The formulated problem was a challenging mixed-integer non-convex problem. To facilitate solving it, we transformed the formulated problem into an equivalent problem with special structure by using some appropriate transformations. On this basis, we devised a low-complexity algorithm with promising performance by using the penalty convex–concave procedure method. Numerical results finally demonstrated the superiority of the proposed solution.

## Data Availability

Not applicable.
